# Integrated Review of Transcriptomic and Proteomic Studies to Understand Molecular Mechanisms of Rice’s Response to Environmental Stresses

**DOI:** 10.3390/biology13090659

**Published:** 2024-08-25

**Authors:** Naveed Aslam, Qinying Li, Sehrish Bashir, Liuzhen Yuan, Lei Qiao, Wenqiang Li

**Affiliations:** State Key Laboratory for Crop Stress Resistance and High-Efficiency Production, College of Life Sciences, Northwest A&F University, Yangling 712100, China; naveedbot197@gmail.com (N.A.); li15725480698@163.com (Q.L.); sehrishbashir88@gmail.com (S.B.); 15934546170@163.com (L.Y.); leiqiao@nwafu.edu.cn (L.Q.)

**Keywords:** rice, transcriptomics, proteomics, environmental stress, molecular mechanism, genetic improvement and breeding

## Abstract

**Simple Summary:**

Rice production is strongly affected by environmental stresses such as drought, heat and salt. Rice plants have developed intricate molecular mechanisms to deal with these challenges. Researchers have identified crucial genes, proteins and metabolic pathways involved in these responses through transcriptomic and proteomic studies. Understanding these molecular mechanisms can help in developing new stress-resistant varieties, thus ensuring stable production under adverse environmental conditions. This review aims to provide a straightforward summary of the progress in understanding the molecular mechanisms of rice’s response to various environmental stresses through transcriptomic and proteomic studies.

**Abstract:**

Rice (*Oryza sativa* L.) is grown nearly worldwide and is a staple food for more than half of the world’s population. With the rise in extreme weather and climate events, there is an urgent need to decode the complex mechanisms of rice’s response to environmental stress and to breed high-yield, high-quality and stress-resistant varieties. Over the past few decades, significant advancements in molecular biology have led to the widespread use of several omics methodologies to study all aspects of plant growth, development and environmental adaptation. Transcriptomics and proteomics have become the most popular techniques used to investigate plants’ stress-responsive mechanisms despite the complexity of the underlying molecular landscapes. This review offers a comprehensive and current summary of how transcriptomics and proteomics together reveal the molecular details of rice’s response to environmental stresses. It also provides a catalog of the current applications of omics in comprehending this imperative crop in relation to stress tolerance improvement and breeding. The evaluation of recent advances in CRISPR/Cas-based genome editing and the application of synthetic biology technologies highlights the possibility of expediting the development of rice cultivars that are resistant to stress and suited to various agroecological environments.

## 1. Introduction

Rice (*Oryza sativa* L.) is the most important human food crop in the world, feeding more than half of the world’s population [1]. As a global food crop, the yield, quality and adaptability of rice cultivars are of great importance for ensuring food security and promoting agricultural development, making these the most important goals for rice breeding and genetic improvement [2,3]. In recent years, with the increase in extreme weather conditions, it has become more and more challenging to make breeding decisions for rice productivity and quality issues [4]. In fact, due to the impact of global climate change and increasing population and industrialization, environmental stress has become one of the main factors restricting crop yield and quality, even though yield in the cultivation of most cereals, including rice, has plateaued in recent decades [2,3]. Climate change and global warming increase the severity, duration and frequency of environmental stresses during rice growing season, threatening agricultural sustainability and global food security in the coming decades [5]. By 2050, global warming and climate change are projected to reduce irrigated rice production by 7%, while rain-fed rice yields may decrease by 6% and potentially up to 2.5% [6].

To cope with the adverse effects of increasing environmental stresses on crop yield and quality, it is necessary to establish more effective breeding and cultivation strategies for the efficiency and sustainability of crop production. In the past few decades, various strategies have been employed in climate-resilient agriculture to ensure the long-term sustainability of rice production. For instance, the “green revolution” led to a substantial increase in rice yield by the development of the high-yield semi-dwarf rice cultivar that saved the world from a chronic food shortage in the 1960s [7]. However, adverse environmental conditions continue to negatively affect rice production, causing significant yield losses. Biotic and abiotic stresses, either individually or in combination, hinder the realization of the full genetic potential for optimal rice yield and productivity [8].

Plants respond to environmental stress through a series of changes at the physiological, biochemical and molecular levels [9,10]. At the molecular level, plants sense and respond to abiotic stresses through the down- or up-regulation of stress-responsive genes and proteins, and these changes can be identified by genome-wide expression analysis by transcriptomic and proteomic methods, including RNA-seq and mass spectrometry-based proteomic methodologies [11]. These high-throughput technologies have enhanced agriculture by deepening our knowledge of complex molecular response mechanisms, differential gene or protein expressions and regulatory pathways under different environmental conditions [12]. In the past twenty years, the elucidation of the molecular basis for important traits in rice and their genetic improvements is one of the most important scientific areas in rice biology. The significant progress in transcriptomics and proteomics has accelerated the discovery and functional characterization of key genes crucial for improving rice productivity and tolerance to various environmental stresses [12,13].

In molecular biology, the word “ome” refers to the study of spatial, temporal and global changes occurring in an organism. The study of omics is a field of biology that aims to understand the functions of cells, tissues and organs, as well as to extract significant biological data [14]. The completion of the rice genome sequence has provided a necessary condition for several omics studies on rice [15,16]. Since then, significant progress has been made in rice genomics, transcriptomics, proteomics, metabolomics, etc., covering almost all aspects of organelles, cells, tissues and organs [17,18,19,20]. To achieve sustainable rice productivity, it is important to understand the molecular mechanism that underlies rice’s response to environmental stresses and the genetic variation that increases rice resistance to adverse environmental conditions. Transcriptomics tracks genome-wide gene expression patterns, while proteomics reveals precise protein abundance and post-translational modifications in responses to various environmental stress [21]. Advanced proteomic techniques have improved experimental methodologies for investigating gene function and phenotypic changes in plant response to biotic and abiotic stresses [22]. By using omics data, we can observe how stress adaptation works on a systemic level, which can help us better understand stress tolerance mechanisms despite the complexity of the underlying molecular landscapes.

**Figure 1:**
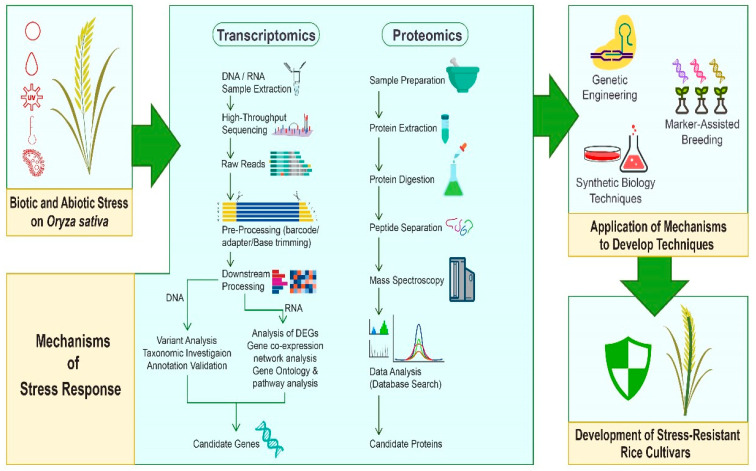
A graphical route for transcriptomic and proteomic strategies to investigate rice’s responses to biotic and abiotic stresses.

This review summarized the research progress and application of transcriptomic and proteomic studies to investigate the molecular mechanisms of abiotic stress tolerance in rice. As shown in Figure 1, a graphical overview for studying rice stress response and molecular mechanisms using transcriptomic and proteomic strategies is provided (Figure 1). Our goal is to consolidate existing knowledge and provide a comprehensive overview of transcriptomics and proteomics to understand rice’s response to abiotic stress and genetic improvements. The insights in this review are significant for developing stress-tolerant rice varieties, ensuring sustainable agriculture and maintaining global food security within growing environmental challenges.

## 2. Brief Overview of Environmental Stress Responses and Mechanisms in Plants

In long-term evolutionary history, plants have evolved sophisticated mechanisms, including a series of physiological, biochemical and molecular responses to deal with or adapt to adverse environmental conditions [9,10]. To ensure their survival and growth in various abiotic stresses like drought, salinity, heat, cold and heavy metal stresses, plants may activate several mechanisms [23]. Phytohormones, especially abscisic acid (ABA), play a crucial role in the physiological, biochemical and molecular responses of plant to various abiotic stresses [24,25]. Physiologically, a common effect of various environmental stresses on plants is the disruption cellular redox state which leads to the excessive accumulation of reactive oxygen species (ROS) and results in damage to cellular components [26,27]. On a molecular level, these abiotic stresses may trigger the reprogramming of the gene expression of plants by transcriptional, translational or post-translational regulations to turn on a variety of defense mechanisms in response to the stresses [28]. These responses ultimately manifest as changes in the relative abundance of proteins and enzyme activities. This can be caused by the upregulation or downregulation of stress-responsive genes and proteins, which alters the overall transcriptome or proteome levels [29].

Additionally, because of the ubiquitous effects of post-transcriptional regulatory mechanisms (e.g., mRNA stability) and post-translational modifications, the expression patterns at the mRNA and protein levels do not always coincide with each other [30]. Furthermore, the complexity of the stress response can be significantly impacted by the extent and duration of different environmental stresses. Phenotypic plasticity, the ability to produce multiple phenotypes from a single genotype, is considered one of the major means by which plants can cope with environmental factor variability. Theoretically, phenotypic plasticity in plants, which is directly associated with the availability of proteins, especially storage proteins, may play a determinant role in plant response to environmental stress conditions [31].

For a comprehensive understanding of the molecular mechanisms that plants use to respond to various abiotic stresses, it is important to study the genes and proteins involved in the transcriptional regulation, biochemical metabolism and signal transduction pathways, especially those related to plant hormone metabolism and signal transduction. In signal transduction cascades, the regulation of stress-responsive genes is mainly attributed to second messenger molecules. It is widely accepted that the transient increase in cytoplasmic calcium (Ca^2+^), acting as the second messenger, plays a crucial role in plant response and tolerance to several types of environmental stresses [32,33,34]. For example, Ca^2+^ signaling leads to the activation and transmission of cold signals through the increase in cytoplasmic Ca^2+^ concentrations which regulate cold-responsive genes such as *OsCDPK-24*, CIPK7 and other responsive genes [35,36]. Moreover, it has been demonstrated that ROS signaling activating Ca^2+^ channels also act as a signaling mechanism in stress responses [37]. The activation of stress-responsive genes subsequently elicits a series of physiological and biochemical responses including increasing superoxide dismutase (SOD) and catalase (CAT) activity to scavenge excess ROS and rebalancing cellular redox status [38,39,40]. Over the past few decades, while significant progress has been made in understanding the signal transduction involved in plant responses to various environmental factors, there are still many unclear aspects of regulatory molecular cascades. With the global climate changing, it will be more and more challenging to determine the mechanisms underlying plant response to environmental stresses from a static perspective. However, transcriptomics and proteomics may provide powerful tools to comprehensively characterize environmental stress responses in rice.

## 3. Transcriptomic Approaches in Studying Rice’s Responses to Environmental Stresses

Among omics technologies, transcriptomics is the most widely used in studying the molecular mechanism of rice’s response to various abiotic stresses. Transcriptomics studies an organism’s entire RNA transcriptome at a particular physiological state or developmental stage [41]. It is possible to link genotypes to certain phenotypes by comparing transcriptomes from different populations. Through transcriptomic analyses, researchers may yield genome-wide gene expression profiles in addition to identifying mRNA, long non-coding RNAs and short RNAs [42]. One strategy includes hybridization-based techniques, such as microarray and suppression subtractive hybridization (SSH), which were commonly used in the early research of transcriptomics. Another strategy consists of sequencing-based transcriptomic techniques, especially RNA-seq which is widely used in biological and biomedical studies. In terms of resolution and coverage, RNA-seq performs better than hybridization and other sequencing methods [43]. In the past two decades, transcriptomic strategies have identified numerous genes responsive to abiotic stresses. The upregulation or downregulation of these genes can directly influence rice tolerance to environmental stresses.

To explore how drought stress affects morphological and transcriptomic changes in rice floral development, microarray analysis was conducted on rice florets of different sizes. This analysis identified over 1000 drought-responsive genes, many of which were specifically regulated in florets of a certain size, indicating that rice responds to drought in a manner dependent on the developmental stage of the florets [44]. Additionally, the analysis revealed that drought stress reprogrammed the pathway related to gibberellin acid GA signaling and ABA catabolism. This suggests that drought stress affects interactions between reproductive development, phytohormone signaling and carbohydrate metabolism in rice plants [44]. High-throughput transcriptomic analysis showed that thousands of genes display differences in expression between upland rice and lowland rice. These differences contribute to the distinct morphological traits related to drought tolerance and adaptation seen in upland rice during its domestication. This provides valuable insights for breeding rice varieties that are more water-efficient and drought-resistant. [45]. Previously, knowledge about the temporal dynamics of rice transcriptomes under drought was limited. Xia et al. (2020) investigated temporal transcriptomic dynamics in 12 rice genotypes and revealed that drought-tolerant genotypes possess less differentially expressed genes (DEGs) but have higher proportions of upregulated DEGs [46]. Recently, a comparative transcriptomic analysis of different rice cultivars from northeast India showed that significant differences in both transcriptional and post-transcriptional regulations shape transcriptome profiles, influencing how each variety responds to drought stress [47]. Similarly, an RNA-seq analysis of rice roots also revealed that post-transcriptional regulation plays a role in how the plant responds to high cadmium stress [48].

To uncover the molecular responses to heat stress in rice floral organs, RNA-seq analysis identified five pectinase genes that show a strong response to heat stress. This finding offers a potential molecular strategy to address heat damage in rice [49]. To identify novel genes and mechanisms related to chilling tolerance responses in rice, transcriptomic analysis was conducted on three cold-tolerant genotypes and one cold-sensitive genotype under both normal and cold stress conditions. This analysis identified 2242 commonly regulated differentially expressed genes DEGs during cold stress in all of the genotypes, with 318 DEGs specifically associated with cold tolerance found in the three cold genotypes [50].

Since the tolerance or sensitivity to abiotic stresses involves a complex interplay between molecular, biochemical and physiological mechanisms, some researchers have conducted meta-analyses of comparative transcriptomic data to identify key genes involved in abiotic stress tolerance in rice [51]. More investigations prefer to integrate transcriptomics and physiology to reveal the mechanisms of plant resistance to various stresses [52]. Recently, a study combined GWAS and transcriptomics to identify candidate genes associated with heat tolerance in rice. This provides valuable candidate genes for improving rice heat tolerance through molecular breeding [53]. Additionally, it was also reported that integrating transcriptomics, gene co-expression networks and gene family characterization is helpful in studying the responses to abiotic stresses in rice [54,55,56].

Although plants can activate various mechanisms to survive and grow under abiotic stress, some studies suggest that different types of environmental stress may activate the same signaling pathway. For example, to understand the interplay between signaling pathways in response to *Xanthomonas oryzae* infection and drought stress, transcriptomic analyses were conducted on H471, a rice introgression line that exhibits broad-spectrum dual resistance to *Xanthomonas oryzae* and drought tolerance [57]. A total of 178 genes that were differentially expressed in H471 were common in response to both stresses when compared with its recurrent parent HHZ. These genes are involved in signaling perception and transduction, transcription regulation and stress response, indicating the overlap between signaling pathways activated by *Xanthomonas oryzae* infection and drought stress [57]. A similar study, through a transcriptomic analysis of *Xanthomonas oryzae* infection and drought stress in different rice genotypes, identified many potential genes, particularly those associated with translation. These genes could be used as molecular markers and targets for genetic manipulation to improve multi-stress tolerance and develop durable resistant rice cultivars [58]. Additionally, comparative transcriptomics of rice plants under cold, iron and salt stresses revealed that 370 differentially expressed genes (DEGs) were common to the three stresses, indicating a significant relationship among these abiotic stresses [59].

## 4. Proteomic Approaches in Studying Responsive Mechanisms to Environmental Stresses

Proteomics deals with functional proteins’ roles, structures, localizations and interactions with other proteins and their implementation in stress or natural conditions. Thus, proteomic studies may identify potential protein markers whose changes in abundance can be associated with quantitative changes in some physiological parameters used to describe the genotype’s level of stress tolerance [60]. To identify key proteins and elucidate their underlying mechanisms of stress response in rice, many investigators have performed comparative proteomic studies on rice varieties with various tolerances or under different stress conditions [22].

Early this century, the strategy of combining two-dimensional gel electrophoresis (2-DE) with mass spectrometry (MS) was widely used to identify stress-related proteins and pathways. For example, the application of 2DE and MS techniques was used to investigate the difference in cold tolerance between two distinct rice cultivars, which identified 59 proteins related to the resistance of cold stress [61]. To study salt stress-responsive proteins in rice, three-week-old seedlings were treated with NaCl, and total proteins in roots were extracted and then separated by 2DE, which led to the identification of 10 salt stress-responsive proteins through MS analysis and database searching [62]. These proteins are involved in the regulation of carbohydrate, nitrogen and energy metabolism, reactive oxygen species scavenging, mRNA, protein processing, and cytoskeleton stability, which gives new insights into salt stress response in rice roots and highlights the effectiveness of the 2DE-based proteomic approach in plant biology studies [62]. To study the initial response of rice to drought stress, two-week-old rice seedlings were subjected to drought conditions, and the changes in protein expression were analyzed using the 2DE approach, which revealed ten drought stress-responsive proteins with increased abundance and two proteins with decreased levels [63]. These proteins are involved in the defense, energy, metabolism, cell structure, and signal transduction pathways [63].

Some proteomic studies investigate the impact of a specific gene’s function on the proteome changes in rice mutant or overexpressing transgenic plants under stress conditions. For instance, to understand the early salt response of rice roots and identify the SnRK2 signaling pathway, proteome changes were analyzed by 2-DE, and protein spots were identified by LC-MS/MS from wild-type and transgenic rice roots overexpressing a rice SnRK2 kinase [64]. A comparative quantitative analysis of the proteome showed that 43 early salt-responsive proteins were more abundant in transgenic rice roots in unstressed conditions, and these proteins contain potential SnRK2 recognition motives. Furthermore, in vitro kinase assay revealed that one of the identified proteins, calreticulin, is a suitable substrate of the SnRK2 kinase [64]. In addition to the above studies, many investigations also used the 2-DE strategy to study the response of rice to various abiotic stresses and identified hundreds of stress-responsive proteins at different developmental stages in rice [65,66,67,68,69,70,71,72,73,74].

In recent years, with significant progress in protein labeling and mass spectrometry technologies, high-throughput proteomic methodologies such as isobaric tags for comparative and absolute quantification (iTRAQ)-based quantitative proteomics have been widely used to analyze the changes in proteomes in plant biology studies [75,76,77,78]. These advanced proteomic technologies exhibit high sensitivity, excellent reproducibility, wide-range, and high-throughput analysis capabilities and other advantages, making them powerful tools to identify and quantify the levels of relevant sets of proteins in various plants [75,76,77].

An iTRAQ-based comparative proteomic study was conducted to investigate the salinity-responsive proteins and related biochemical features of two contrasting rice genotypes, which identified a total of 5340 proteins in both rice genotypes [79]. Functional characterization by KEGG, COG, and GO enrichment results suggest that differentially expressed proteins are mainly involved in the regulation of salt stress responses, oxidation–reduction responses, photosynthesis, and carbohydrate metabolism [79]. To investigate the salt-tolerant mechanism of a rice mutant with a novel allele of heterotrimeric G protein alpha subunit (RGA1), the iTRAQ-based proteomic technique identified 332 differentially responsive proteins in seedlings of the mutant and wild type in response to salt treatment [80]. Pathway enrichment analysis revealed that these proteins were mainly involved in the regulation of processes such as metabolic pathways, photosynthesis, and reactive oxygen species (ROS) homeostasis, which is consistent with the lower ROS accumulation and higher ROS scavenging enzyme activities in the RGA mutant, which contributes to the enhanced salt-tolerance in the mutant [80]. The iTRAQ-based quantitative proteomics of rice roots in response to aluminum (Al) stress identified a total of 700 distinct proteins; among them, 106 proteins were differentially expressed in response to Al toxicity in sensitive and tolerant rice cultivars, which may lead to a better understanding of Al tolerance mechanisms in rice and help to improve plant performance in acidic soils [81]. 

Moreover, iTRAQ-based proteomics analysis of endoplasmic reticulum (ER)-stressed rice seeds identified 140 upregulated and 265 downregulated proteins, and further analysis showed that the upregulated proteins were mainly involved in protein modification, transport, and degradation, and the downregulated proteins were mainly involved in metabolism and stress/defense responses [82]. To better understand the molecular mechanism underlying silicon (Si) alleviating cadmium (Cd) stress in rice plants, 100 proteins differentially regulated by Si under short- or long-term Cd stress were identified in iTRAQ-based proteomic analysis [83]. Interestingly, 70% of the proteins were downregulated, suggesting that Si may improve protein use efficiency by maintaining cells in the normal physiological state [83]. Besides these studies, many investigations have also combined iTRAQ-based proteomic strategies to study the molecular mechanisms of rice’s response to various environmental stresses, such as salt [84,85,86,87], high temperature [88,89], low temperature [90,91], cadmium (Cd) [92,93,94], zinc (Zn) [92], nitrogen (N) deficiency [95], low light [96], *Magnaporthe oryzae* infection [97], etc.

In addition to iTRAQ-based proteomic strategies, other high-throughput proteomic methods have also been used to study the molecular mechanisms of rice’s response to different stresses. For instance, a gel-free/label-free proteomic technique was applied to analyze the proteome changes in developing grains of rice cultivar Khao Dawk Mali 105 under heat stress [98]. It revealed that the proteins involved in glycolysis and the tricarboxylic acid cycle and redox homeostasis were disrupted by heat stress, suggesting that proteins involved in redox homeostasis and the carbohydrate pathway may play an important role in rice grain’s response to heat stress [98]. A label-free quantitative shotgun proteome analysis was also performed to study the response of different rice genotypes in well-watered and drought conditions. These proteomic investigations identified many key proteins and stress-responsive pathways. Exploring these protein functions will enable a deep understanding of the complex molecular mechanisms and regulatory signaling networks underlying rice’s response to various environmental stresses. In short, these transcriptomic investigations have enabled a deep understanding of the complex molecular mechanisms and signaling regulatory networks underlying rice’s response to various environmental stresses.

## 5. Integrating Multi-Omics Strategies for Rice’s Responses to Environmental Stresses

With increased difficulty and depth in plant research programs, the limitations and drawbacks of using a single omics to study plant signal transduction and molecular mechanisms have gradually become apparent in recent years. Due to the technological advancements, and decreased costs in genomics, transcriptomics, proteomics and metabolomics, multi-omics-based research approaches are becoming more and more popular. Integrated multi-omics datasets have been effective tools for exploring the essential genes, proteins signaling pathways and other small molecules associated with abiotic stress responses.

Over the past few decades, many researchers have integrated transcriptomic and metabolomic strategies to study the molecular responses of rice to drought stress [99,100,101,102]. A systematic study of rice floral organ responses to heat and drought stresses was conducted by combining metabolomics with transcriptomic changes, which revealed sugar starvation as a factor in reproductive failure under these stresses [99]. Integrated transcriptomic and metabolomic studies have identified the key metabolic pathways that support well-maintained photosynthesis under drought conditions and lead to improve drought tolerance in rice [100]. Morphological, transcriptomic, and proteomic analyses of the responses of drought-tolerant and -sensitive rice varieties have shown that the expression of the Med37c and RSOsPR10 genes varies among different rice varieties, while chitinases may play a significant role in drought tolerance [103]. A comprehensive metabolomic and proteomic investigation in the biochemical metabolic pathways of rice spikes under drought and submergence stress has revealed that the two stresses mainly promote the energy metabolism pathway, carbon fixation in photosynthetic organism pathway, carbohydrate metabolic process and reactive oxygen species (ROS) metabolic process functions [104]. A combined analysis of rice growth and grain yield through proteomics, metabolomics, and physiological methods, under the conditions of heavy nitrogen application before and after drought, suggests that applying heavy nitrogen application before drought can be a key strategy to enhance rice yield and stress resistance. This approach offers a new ecological perspective on nitrogen regulation in rice [105]. Analyzing the effects of space flight on rice progeny through combining proteomics and metabolomics analysis has shown that oxidative stress signals trigger sugar signals to rebuild metabolic networks to adapt to space flight stress. The reconstruction of energy metabolism, amino acid metabolism, phenylalanine metabolism and flavonoid metabolism plays an important role in the process of adapting to space flight stress [106]. To understand rice’s response and tolerance to low and high temperature, integrated transcriptomic and proteomic approaches have shed light on the molecular mechanisms of cold tolerance [107] and heat tolerance [108] in rice.

Recently, besides multi-omics strategies, some investigations have also combined transcriptomics with other molecular approaches, such as gene family characterization [56], gene co-expression networks [55], genome-wide association (GWAS) [53,109] and ATAC-seq [110], to analyze rice’s response and tolerance to various environmental stresses. For example, to identify new candidate genes, *LOC_Os01g04814*, which encodes a vacuolar protein sorting-associated protein 4B, and *LOC_Os01g04814*, which encodes a glycosyltransferase family 43 protein, are used. These genes are responsible for cold stress and chilling acclimation in rice [109]. To identify new loci and favorable alleles associated with heat tolerance in rice, an integrated analysis of GWAS and transcriptomics identified *LOC_Os07g48710* as a promising candidate gene. This gene may provide valuable insights for enhancing rice heat tolerance through molecular breeding [53].

Moreover, the rapid development of mass spectrometry and DNA sequencing technologies has made it possible to detect subtle changes in the transcriptome and proteome in a single cell, just as its name implies, single-cell omics. Recent advances in single-cell proteomics for animal systems have been adapted for plants to increase our understanding of plant development, response to stimuli, and cell-to-cell signaling [111,112]. Single-cell sequencing approaches can reveal the intracellular dynamics of individual cells, and elucidate biological mechanisms with high-dimensional catalogs of millions of cells, including genomic, transcriptomic, chromatin accessibility, epigenomic and proteomic data across species [113,114]. Now, they have entered the stage of combining single-cell proteomic and transcriptomic, spatial transcriptomic, and spatial metabolomic techniques to analyze plant development and environmental responses [115,116,117,118]. This will provide revolutionary new insights for the understanding of the mechanisms underlying rice’s response to environmental stress.

## 6. Genetic Improvements and Breeding for Rice Tolerance

Sustainable crop production is a major challenge in today’s global climate change scenario. In recent years, significant progress in rice omics has greatly enhanced our understanding of various stress responses in rice. Insights from these omics analyses have enabled the identification of candidate genes that can be used for plant breeding. In such a background, some studies have provided comprehensive overviews of all detected protein changes involved in drought stress, aiming to define new genetic markers for marker-assisted selection breeding [119]. For example, an integrated metabolomic and proteomic study of rice spikes under drought and submergence stress revealed the key metabolic pathway which provides the genetic basis for the breeding of drought- and submergence-resistant varieties [104]. The combination of genomics and transcriptomics identified candidate loci related to cold tolerance in Dongxiang wild rice, which also provided insights into the genetic improvement in cold stress in rice breeding programs [120].

The advent of gene editing technologies such as the CRISPR-Cas9 genome editing system has opened new opportunities for advancing crop genetic improvements and breeding [121]. These technologies enable the precise editing of target genes, which helps create higher-quality and more disease-resistant varieties. CRISPR/Cas-mediated genome-wide screens make it possible to discover novel traits, expand the range of traits and speed up trait development in target plants, which have revolutionized biological research and its applications in crop improvement [122]. Recent advances in CRISPR/Cas genome editing have made it possible to efficiently target, and modify genes in most crops, offering great potential to accelerate crop improvement [123,124]. In crop breeding programs, the CRISPR/Cas system has been considered a versatile tool for editing in all layers of the central dogma with a focus on the development of plants resistant to multiple biotic and abiotic stresses [125,126]. CRISPR-Cas9 genome engineering could be used to improve salt stress tolerance in rice by targeting key genes such as NHX and SOS1 transporters [127]. Reports indicate that rice yield and cold tolerance can be effectively enhanced by using the CRISPR/Cas9 system to edit genes such as *OsPIN5b*, *GS3* and *OsMYB30* [128]. Rice salinity tolerance can be improved through the CRISPR/Cas9-targeted mutagenesis of the *OsRR22* gene [129]. The CRISPR-Cas9 mediated genome editing of the *OsDST* gene significantly improved drought and salt tolerance and grain yield in rice cultivar MTU1010 [130].

A recent study generated CRISPR/Cas9-induced mutations in the OsDSG1 gene of a local rice cultivar to develop and use salt-tolerant rice varieties. This approach improved salt tolerance at both the germination and seedling stages, offering a promising strategy to enhance salt stress resistance in local rice varieties [131]. Since environmental stresses such as salinity significantly affect rice at both the seedling and reproductive stages, recent advancements in CRISPR technology, including targeted mutagenesis and recent prime editing, can better help in gene discovery and functional analysis as well as in transferring favorable alleles into elite breeding material to speed up the breeding of tolerant rice cultivars [132,133]. Using CRISPR-mediated base editors, it is reported that the base-editing-mediated artificial evolution (BEMG) of *OsALS1* in planta can be used to develop novel herbicide-tolerant rice germplasms, indicating the great potential of BEMGE in creating important genetic variants of target genes for crop improvement [134]. Moreover, previous research has shown that the CRISPR/Cas9 editing of SERK2 can regulate salt tolerance. This highlights the significant potential of targeting specific BR components, such as SERK2, for crop improvement through flexible strategies [135].

Recently, it has been demonstrated that the integration of CRISPR gene editing technology and plant synthetic biology will be a cutting-edge technique for the next stage of crop genetic improvement [136,137]. Photosynthetic efficiency is relevant to sustainability and food security because it affects water use efficiency, nutrient use efficiency and land use efficiency. Some studies specifically focus on the potential of synthetic biology approaches for improving photosynthesis to drive a sustainable increase in crop yields [138,139]. These studies will provide us with new perspectives for systematically improving multi-stress resistance in rice. The application of multi-omics and big data analysis has advanced the development of more effective genetic improvements in crops, and some researchers suggest enhancing crop temperature resilience by integrating omics data with systemic and synthetic biology, especially through molecular module programs [140]. As the “chips” of agriculture, seeds are crucial to agricultural sustainability and global food security. The core goals for crop improvements and breeding in future agriculture will be highly dependent on the superior quality of seeds, which may reduce the use of chemical fertilizers and pesticides and decrease yield losses caused by global climate change, extreme weather conditions and other environmental stresses. In this regard, breeding technologies should adopt more cutting-edge sciences such as multi-omics, gene editing and synthetic biology approaches, machine learning and advanced statistical models, enabling predictive breeding [141].

## 7. Future Prospects and Hot Topics

To cope with adverse environmental conditions, plants may activate a series of physiological, biochemical and molecular responses to ensure their survival, and growth. These responses ultimately manifest as changes in the abundance of transcripts, proteins, metabolites, and other molecules. These changes can be caused by an increase or decrease in stress-responsive genes and proteins, changing the transcriptome and proteome levels overall. Towards a deep understanding of the responsive mechanisms of rice dealing with environmental stresses, the large-scale identification of stress-responsive genes and proteins is of great importance for elucidating the molecular mechanisms in rice. In this respect, many omics investigations in rice identified a great number of responsive genes, proteins, and pathways, which will provide not only a systematic understanding of stress-responsive mechanisms but also an omics perspective for the genetic improvement and breeding of rice cultivars with ideal resistance.

In the history of world agriculture, plant breeding over the centuries has gone through three stages [142]. The first stage is selection breeding, which relies on natural populations containing spontaneous variation to cultivate crop varieties with specific environmental adaptability. The second stage is hybridization breeding, based on the crossing of different genotypes followed by trait selection [142]. Hybridization breeding relies mainly on the theories, and applications of statistics, quantitative genetics, heterosis and mutagenesis breeding, which has become a common practice in plant breeding. The third stage is molecular breeding, which is mainly based on modern molecular biology strategies such as DNA recombination and sequencing, molecular markers (i.e., marker-assisted breeding) and transgenic technologies [142].

We are entering a new revolutionary era, which will be intelligent breeding or digital breeding. This approach combines multidimensional omics, gene editing, big data and artificial intelligence (AI) large language models to design and breed desirable cultivars. Intelligent breeding aims to develop predictive breeding methods at a higher level to minimize human intervention by automatically proceeding breeding design and propagating breeding populations and to make selections that account for various environments and climates throughout the breeding process [141].

With the increase in global climate change, agricultural production, and sustainable development are facing increasing challenges. Global climate change, and warming have led to the increased severity, duration, and frequency of environmental stresses on crop production, threatening agricultural sustainability and global food security in the coming decades [5]. In recent years, rice multi-omics studies have identified a large number of stress-responsive genes, proteins and pathways, which will ultimately serve rice tolerance improvement and breeding. However, to achieve the goals of multi-resistance, high yield and high quality in rice, it is not only necessary to integrate functional genes related to yield, quality and resistance with omics big data and AI big prediction models but to also comprehensively utilize gene editing and synthetic biology strategies to achieve genome artificial intelligence precision breeding.

## Data Availability

The data presented in this study are available in this article.
